# Papillary renal cell carcinoma with extensive spindle cell foci: mimicker of mucinous tubular and spindle cell carcinoma

**DOI:** 10.4322/acr.2024.479

**Published:** 2024-03-05

**Authors:** Fareed Rajack, Shawn Medford, Tammey Naab

**Affiliations:** 1 Howard University Hospital, Department of Pathology and Laboratory Medicine, Washington, D.C., United States of America; 2 Howard University College of Medicine, Washington, D.C., United States of America

**Keywords:** Carcinoma, Papillary, Hippo Signaling Pathway, Immunohistochemistry, Kidney Neoplasms, Nephrectomy

## Abstract

Papillary renal cell carcinoma (PRCC) is the second most common renal cell carcinoma (RCC), accounting for 10-15% of cases. Mucinous tubular and spindle cell carcinoma (MTSCC), on the other hand, accounts for only 1% of renal tumors and has a more favorable prognosis compared to PRCC. We report a 75-year-old female with a left upper pole solid renal mass displaying features of both papillary renal cell carcinoma (PRCC) and mucinous tubular and spindle cell carcinoma (MTSC). In this case, a shaggy luminal surface, multiple papillations, and psammoma bodies, absence of E-cadherin expression, and strong CD10 expression favored PRCC. Both immunohistochemistry and genomic analysis are critical to diagnose and differentiate tumors that may have overlapping features accurately.

## INTRODUCTION

Papillary Renal Cell Carcinoma (PRCC), the 2nd most common RCC, accounts for 10-15% of cases and is usually composed of tubules and papillae with foamy histiocytes in papillary cores. Mucinous tubular and spindle cell carcinoma (MTSCC) is composed of tightly packed, elongated, curvilinear tubules with smooth luminal surfaces separated by mucinous stroma. MTSCC is associated with a more favorable prognosis than PRCC.^1^ PRCC and MTSCC have histologic and histochemical overlaps, including elongated tubules and stromal Alcian blue-positive mucin deposits.

PRCC is found more frequently in males, with a male-to-female ratio of 1.5-2:1. It most commonly occurs between the 6^th^ and 8^th^ decades of life. The incidence is almost three times greater in black than in white patients.^2,3^ The most common sites of metastases are lung, bone, liver, and brain.^2^

MTSCC was originally described in 1997 and was categorized under “low-grade collecting duct carcinoma,” along with tubulocystic carcinoma.^1,4^ Its current name was established in 2004 by the WHO’s Renal Tumor Classification Committee. It is a rare neoplasm, accounting for <1% of the renal tumors.^5^ MTSCC is more commonly found in females with a female-to-male ratio between 3-4:1.^1^ While it demonstrates indolent behavior, it is crucial to rule out sarcomatoid carcinoma, another neoplasm with spindle cell features, and a much worse prognosis.^6^ Aside from PRCC and sarcomatoid RCC, other differential diagnoses with similar features include mesenchymal tumors (leiomyoma, acute myeloid leukemia, Inflammatory myofibroblastic tumor, and Juxtaglomerular Cell Tumor) and metanephric adenoma. Tumors under 5 cm are generally homogenous compared to those over 5 cm, which are usually heterogenous.^5^

MTSCC is most commonly found in the renal cortex and in rare cases in the renal medulla.^5,7^ While studies suggest the loop of Henle as a possible source, the origin of MTSCC is still uncertain.^8^ Most cases are incidental findings from abdominal imaging. A hypovascular pattern can be observed on both ultrasound and CT with contrast enhancement, similar to papillary RCC and chromophobe RCC.^5^

## CASE REPORT

**Figure 1:**
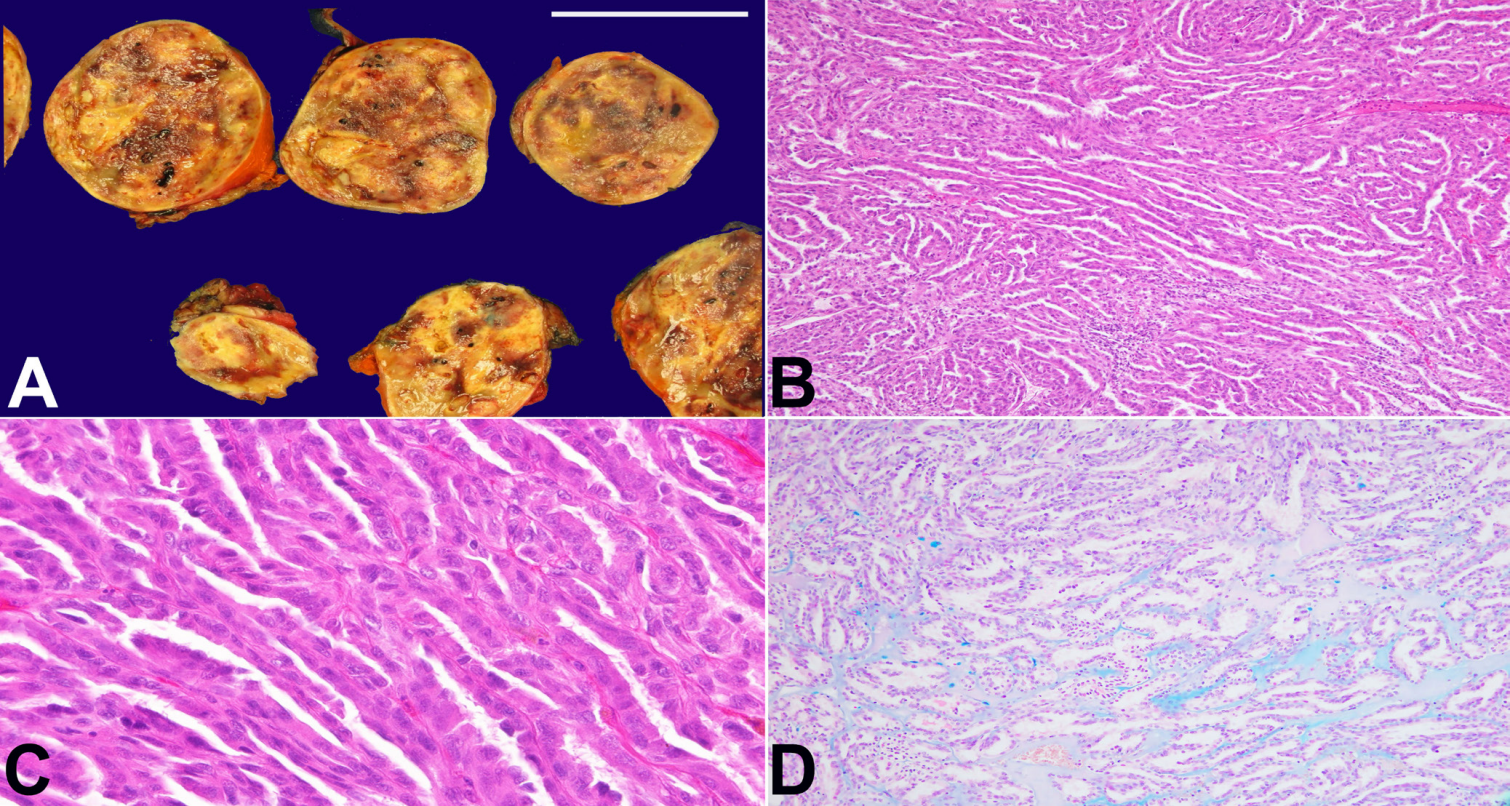
**A -** Gross pathologic examination of the cut surface with areas of hemorrhage and necrosis (sacle bar= 5 cm); **B, C** and **D -** photomicrographs of the tumor; **B -** displays spindle cell change with elongated tubules reminiscent of MTSCC (H&E, 100X); **C -** displays shaggy luminal surface (H&E, 400X); **D -** displays Alcian blue positive mucinous stroma (Alcian blue, 100X).

We report a case of a 75-year-old female who underwent a robotic-assisted partial nephrectomy for resection of a 5.7 x 5.2 x 5.0 cm left upper pole solid renal mass. Grossly, the mass was tan and well-circumscribed with hemorrhagic and necrotic foci (Figure 1A). A wide range of microscopic features were found. Spindle cell change with elongated tubules reminiscent of MTSCC was present in several blocks (Figure 1B); however, the luminal surface was shaggy, favoring PRCC (Figure 1C). Patchy prominent extracellular Alcian blue positive mucin deposits were also present (Figure 1D).

**Figure 2:**
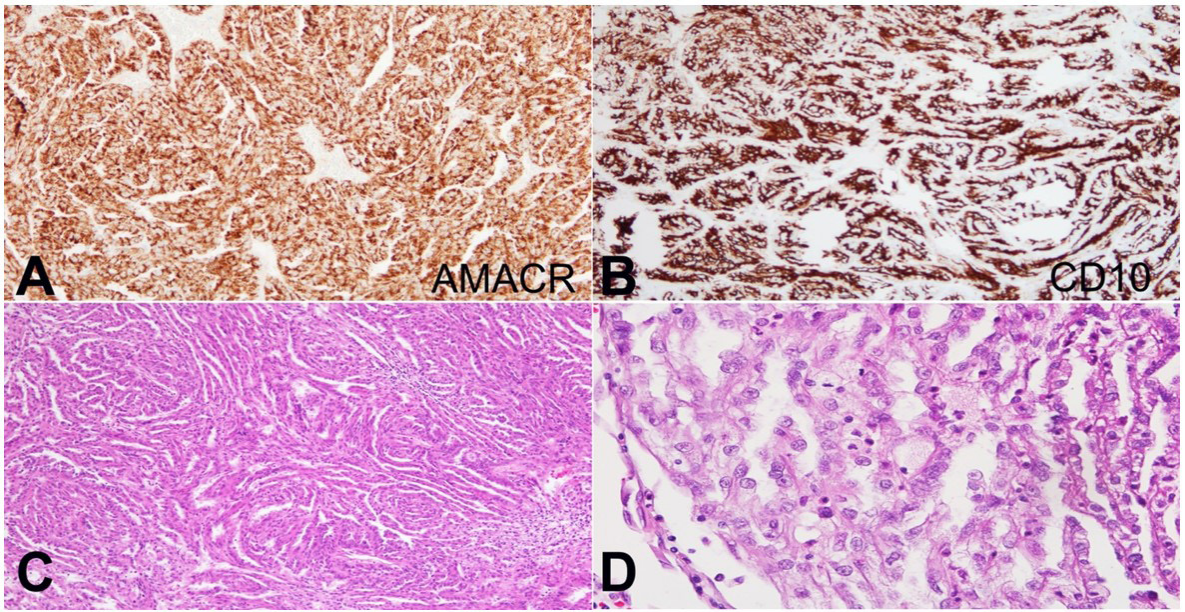
Photomicrographs of the tumor. **A -** displays an AMACR positive stain (100X); **B -** displays a CD10 positive stain, favoring PRCC (immunohistochemistry, low power, 100X); **C -** displays solid spindle cells in a whorled pattern (H&E, 100X); **D -** displays prominent nucleoli in whorled foci (H&E, 400X).

PRCC and MTSCC express CK7, AMACR (Figure 2A), and EMA.^1^ However, the absence of expression of E-cadherin and strong CD10 expression favored PRCC (Figure 2B). Multiple foci of solid spindle cells in a whorled pattern with clear cell change, necrosis, and high-grade nuclei bordering on sarcomatoid RCC were present in other blocks (Figure 2C).^1^ Multiple papillations and psammoma bodies also supported PRCC. A spectrum of spindle cell change was present, ranging from elongated tubules reminiscent of MTSCC to whorled foci with high-grade nuclei approaching sarcomatoid RCC (Figure 2D).

## DISCUSSION

The important morphologic characteristic distinguishing these two entities is the histomorphologic nuclear features of MTSCC versus PRCC. MTSCC has spindle cell foci with low grade nuclei while high grade prominent nucleoli characterize spindle cell change in PRCC.^6^ Therefore, this tumor may be a more aggressive lesion, and close follow-up is recommended.

PRCC and MTSCC may share histologic spindle cell change with elongated tubules and mucinous stroma having Alcian blue positivity. Even papillae with foam cells may be observed in both; however, papillations should not be extensive in MTSCC. Hemorrhage and necrosis rarely occur in MTSCC except in a few cases of sarcomatoid transformation.^1^

Ren et al.^1^ conducted a retrospective study of 26 patients with either MTSCC (11 patients), type 1 PRCC (6 patients), or indeterminate histology with overlapping features (9 patients). They compared morphologic features, immunohistochemistry, and DNA copy number alterations between the three groups. The study identified three features distinguished PRCC from MTSCC: 1) “well-formed, type 1 papillae” in PRCC (40% of cases) vs. small, isolated papillations in MTSCC, 2) “low-grade spindle cell foci” with “spindled tumor cells lining angulated, curvilinear tubules with irregular and shaggy lumina” in PRCC (40% of cases) vs. smoother tubule lumina in MTSCC, and 3) micronodules with small branching papillae containing fibrovascular cores in PRCC (15% of cases). In contrast, the two subtypes could not be differentiated by the presence of a capsule or pseudocapsule, foamy macrophages, mucin, or the percentage of certain architectural features (elongated tubules, short tubules, spindle cells, solid sheets, micronodules/abortive papillae, and well-formed papillae).^1^

Genomic findings are very useful in differentiating these two entities. Trisomy 7, trisomy 17, and loss of the Y chromosome are classic findings in PRCC,^4^ while hypodiploidy with losses of multiple chromosomes is the main finding in MTSCC.^1,9^

Whole exome sequencing of 22 MTSCC cases revealed biallelic loss and/or altered regulation of tumor suppressor genes in the hippo tumor pathway (thought to be disrupted in MTSCC) in 85% of cases.^8^ This most commonly involved *PTPN14* (31%) and *NF2* (22%). YAP and TAZ are two downstream effectors of the pathway.^1^ YAP1 protein expression was increased in 90% of the MTSCC cases. For MTSCC, monosomy was seen in chromosomes 1, 6, 9, 14, 15, and 22 (100% of cases), as well as chromosomes 4 (90% of cases), 8 (81% of cases), 13 (90% of cases).^8^

Ren et al.^1^ analyzed DNA samples using an SNP array platform. PRCC showed chromosomal gains most frequently in chromosomes 7, 16, 17, and 20 and less frequently in chromosomes 2, 3, 10, 12, and 21. MTSCC showed chromosomal copy number losses most frequently in chromosomes 1, 4, 6, 8, 9, 13, 14, 15, and 22 and less often in 10, 18, 11 or 11q. The indeterminate group with overlapping features showed chromosomal gains most frequently in chromosomes 7, 16, 17, and 20. For heterogenous tumors, even the regions of distinct morphology showed either the same or similar copy number alteration patterns.^1^

PAX8, AMACR, CK7, and CAM5.2 expression are found in both PRCC and MTSCC. Strong diffuse CD10 positivity and absent E-cadherin favor PRCC. Ren et al.^1^ immunohistochemistry analysis showed that the greatest difference was seen with CD10, as immunoreactivity was seen in 69% of PRCC cases and 30% of MTSCC cases. This demonstrates a limitation of the use of C10 for cases with indeterminate histologic features. CK7, AMACR, and PAX8 were positive in 100% of cases of both PRCC and MTSCC. CD15 was positive in 70% of PRCC and 60% of MTSCC, and EMA was positive in 80% of PRCC and 90% of MTSCC. YAP and TAZ are two downstream effectors of the Hippo pathway, which is thought to be disrupted in MTSCC. There no significant difference in YAP/TAZ expression between PRCC and PTSCC via immunohistochemical staining.^1^

Chromosomal losses are commonly attributed signatures of MTSCC and likely affect its gene expression. However, the precise mechanism is still not known. More insight into the Hippo pathway’s effects could be gained by having an MTSCC cell line model.^8^ Postulated targeted therapeutics include small molecule inhibitors of *YAP* for patients with sarcomatoid differentiation or metastasis.^8,10^

## CONCLUSION

Submission of multiple sections and awareness of the histomorphologic features of PRCC are essential in making the correct diagnosis. Genomic findings are most helpful in confirming the diagnosis of PRCC. Genomic sequencing may also be useful in differentiating PRCC from MTSCC.
